# Targeting NKG2DLs with an ADCC enhanced fusion protein for induction of NK cell reactivity against ovarian cancer

**DOI:** 10.1186/s13048-025-01962-2

**Published:** 2026-01-21

**Authors:** Ilona Hagelstein, Kevin Wang, Annika Hölz, Johanna Blessing, Martina S. Lutz

**Affiliations:** 1 German Cancer Consortium (DKTK), partner site Tübingen, a partnership between DKFZ and University Hospital Tübingen, Tübingen, Germany; 2 Department of Internal Medicine, Clinical Collaboration Unit Translational Immunology, University Hospital Tübingen, Tübingen, Germany; 3 Cluster of Excellence iFIT (EXC 2180) “Image-Guided and Functionally Instructed Tumor Therapies”, University of Tübingen, Tübingen, Germany

**Keywords:** Immunotherapy, NK cells, Fusion protein, NKG2D, NKG2DL, Ovarian cancer

## Abstract

Ligands for the activating immune receptor NKG2D (NKG2DLs) are frequently overexpressed on malignant cells while largely absent in healthy tissues. Elevated expression of various NKG2DLs has been reported in ovarian cancer, one of the most lethal gynecologic malignancies due to chemoresistance and relapse. Despite the advent of monoclonal antibodies (mAbs) in cancer therapy, patients with ovarian cancer have yet to benefit from this treatment modality. Natural killer (NK) cells play a crucial role in the efficacy of antitumor mAbs by mediating antibody-dependent cellular cytotoxicity (ADCC). Strategies to enhance ADCC often involve Fc region modifications to improve CD16 binding and NK cell activation.

The tumor-associated expression of NKG2DLs was exploited by developing an Fc-optimized NKG2D-Ig fusion protein (NKG2D-ADCC) to trigger NK cell ADCC against ovarian cancer cells. The NKG2D-Ig fusion protein with a wildtype Fc version (NKG2D-WT) was modified in the Fc part by introducing the S239D/I332E mutations, thereby enhancing the binding affinity to the Fc receptor CD16. Flow cytometric analysis revealed a diverse range of expression patterns for NKG2DLs across ovarian cancer cell lines. Functionally, NKG2D-ADCC induced stronger NK cell activation than NKG2D-WT, promoting degranulation and secretion of effector molecules, including IFN-γ and granzymes. The unrelated control protein lacked such effects, confirming specificity. Notably, NKG2D-ADCC induced robust NK cell-mediated lysis of ovarian cancer cells in both short- and long-term cytotoxicity assays.

Together, these findings demonstrate that the NKG2D-ADCC fusion protein potently enhances NK cell responses against ovarian cancer cells, supporting its promise as a novel immunotherapeutic strategy.

**Supplementary Information:**

The online version contains supplementary material available at 10.1186/s13048-025-01962-2.

## Introduction

The immune system plays a critical role in the recognition and elimination of malignant cells. A pivotal mechanism by which the immune system detects and responds to cancer cells involves the activation of immune receptors by stress-induced ligands. The natural killer group 2 member D (NKG2D; CD314), a C-type lectin-like receptor, is - among many other mechanisms - an essential component of this immune surveillance system [1, 2]. NKG2D is expressed on natural killer (NK) cells, T cells, and other immune cell types [3, 4], where it recognizes “induced-self” proteins that are often upregulated in response to cellular stress, including malignant transformation [5, 6]. These proteins, collectively known as NKG2D ligands (NKG2DLs), are typically absent on healthy cells but are induced upon various stressors, such as DNA damage, infection or cancerous transformation [7]. In humans, NKG2DLs consist of major histocompatibility complex (MHC) class I-related chain (MIC) A and B, as well as the UL16-binding proteins (ULBP) 1–6 [8]. Furthermore, NKG2DLs exhibit a high degree of diversity, with more than 100 distinct alleles for MICA, approximately 40 for MICB, and fewer than 20 for the ULBPs being documented [1, 9]. A distinguishing characteristic of these molecules is that, despite their heterogeneity, they are all recognized by the immune system via the NKG2D receptor, thereby inducing an immune response.

These ligands are expressed at high levels on tumor cells across a wide variety of malignancies, including breast, ovarian, lung, and acute myeloid leukemia (AML), among others [10–13]. In addition, elevated levels of NKG2DLs expression have been associated with a more unfavorable prognosis in ovarian cancer patients [14]. Notably, the expression of NKG2DLs can be induced by chemotherapy and radiotherapy, rendering them promising therapeutic targets [15].

The high prevalence of NKG2DLs expression on malignant cells, in conjunction with the tumor-restricted pattern of this expression, has led to the emergence of the NKG2D/NKG2DLs system as an attractive therapeutic target for cancer immunotherapy [16]. Significant efforts have been directed toward the development of NKG2D-based therapeutics [17]. The employment of the NKG2D receptor for targeting NKG2DLs provides a range of benefits when compared to antibody-based methods, which are constrained to a single NKG2DL. The NKG2D receptor exhibits the capacity to recognize a diverse array of structurally disparate ligands, albeit with disparate binding affinities. Examples of ongoing investigations include the use of NKG2D chimeric antigen receptor (CAR) NK cells for the treatment of gastric cancer and multiple myeloma [18, 19], and NKG2D CAR T cells for ovarian cancer [20]. A recent preclinical study investigated the use of a NKG2D bispecific T cell engager as a treatment for glioblastoma [21]. In contrast to these cell-based approaches, recombinant NKG2D fusion proteins provide the advantages of controlled pharmacokinetics, lower toxicity risk and more predictable on-target activity. As off-the-shelf biologics, they offer immediate availability without the need for patient-specific manufacturing, making them a practical and clinically attractive strategy for targeting NKG2DLs expressing tumors.

An engineered NKG2D-Ig fusion protein, incorporating the extracellular domain of NKG2D and the Fc portion of human IgG (NKG2D-WT), has been preclinically explored as a promising candidate for treatment of leukemia and breast cancer [22, 23]. In order to enhance the efficacy of the antibody, amino acid modifications S293D/I332E (SDIE) were introduced in the Fc domain. These modifications augmented the antibody’s affinity for the Fc receptor CD16 (NKG2D-ADCC), thereby amplifying antibody-dependent cellular cytotoxicity (ADCC) [24]. We selected the SDIE Fc modification based on our extensive experience with SDIE engineered antibodies, including the clinically tested FLT3 directed antibody FLYSYN (NCT02789254) [25], which provides a strong translational rationale for applying this established platform to the NKG2D fusion protein. In humans, NK cells predominate as the mediators of ADCC [26]. These cytotoxic lymphocytes play a major role in antitumor immunity mediated by monoclonal antibodies (mAbs). Upon activation, NK cells execute two primary functions. Firstly, they directly lyse tumor cells by releasing cytolytic granules containing proteins such as perforin and granzyme. Secondly, they modulate the immune system by releasing cytokines such as IFN-γ, which in turn amplifies antitumor immune responses [27]. Consequently, we hypothesize that NK cells are particularly well-suited to function as effector cells in novel immunotherapeutic strategies.

Despite recent advancements in immunotherapy for other solid tumors, the prognosis for patients diagnosed with ovarian cancer remains poor. The absence of early symptoms in many patients results in the diagnosis being made at an advanced stage. The therapeutic approach for ovarian cancer encompasses surgical intervention aimed at extirpating the tumor, complemented by adjuvant chemotherapy [28]. Patients in the advanced stage of the disease are being treated with bevacizumab, an mAb that targets vascular endothelial growth factor (VEGF). Patients with a mutation in the *BRCA1/2* gene or another homologous recombination deficiency (HRD) receive maintenance therapy using a poly (ADP-ribose) polymerase (PARP) inhibitor [29]. However, despite these advancements, approximately 70% of patients experience recurrence within 18 to 28 months after initial treatment [30], emphasizing the need for novel treatment options.

Several studies have shown that NK cells infiltrate ovarian carcinoma and can contribute to antitumor activity, and higher NK cell abundance has been associated with improved patient outcomes, indicating that NK cell mediated mechanisms are relevant in this disease [31]. Building on this, recent reviews have emphasized the potential of NK cell-based immunotherapies for ovarian cancer, as relapse rates remain high despite multimodal treatment strategies. Although NK cells are capable of mediating potent antitumor functions such as ADCC and perforin and granzyme dependent cytotoxicity, their activity in solid tumors is often limited by insufficient persistence and the suppressive tumor microenvironment [32]. These insights support the rationale for evaluating ADCC optimized constructs such as the NKG2D-ADCC fusion protein in ovarian cancer, where an unmet clinical need persists and NK cell-based immunity plays an important role.

Preclinical studies have indicated that ovarian cancer is susceptible to attack by NK cells [33–35], underlining the potential of ADCC inducing mAbs for ovarian cancer treatment. In light of this, the present study evaluated the optimized NKG2D-ADCC fusion protein as a potential therapeutic compound for ovarian cancer, a malignancy that is frequently accompanied by robust and prevalent expression of NKG2DLs [31]. The objective of this study was to elucidate the preclinical efficacy of the NKG2D-ADCC fusion protein in targeting ovarian cancer and its potential as a novel immunotherapeutic modality.

## Materials and methods

### Generation of fusion proteins

The fusion proteins targeting NKG2DLs were generated by fusing the extracellular domain of NKG2D (F78-V216) to the C-terminus of an hIgG1 Fc domain, either with a wild-type Fc domain (NKG2D-WT) or with the S239D/I332E mutations (NKG2D-ADCC) [36]. For all assays involving NKG2D-ADCC, the corresponding isotype control consisted of the MOPC-SDIE construct, which carries the same human IgG1 Fc region including the S239D/I332E amino acid modifications but lacks NKG2DLs specificity. This control was used consistently throughout the study, as it represents the most stringent format matched comparator for assessing the specificity and Fc driven activity of NKG2D-ADCC [37].

For expressing the recombinant constructs in ExpiCHO cells (Gibco, Carlsbad, CA), plasmid DNA was isolated using the EndoFree Plasmid Maxi Kit (Qiagen, Hilden, Germany). Antibody purification was carried out using protein A affinity chromatography (GE Healthcare, Chicago, IL), followed by preparative size exclusion chromatography on a HiLoad 16/60 Superdex 200 column (GE Healthcare).

To assess the purity and structural integrity of the purified antibodies, analytical size exclusion chromatography was performed using a Superdex 200 Increase 10/300 GL column (GE Healthcare). In parallel, protein purity was evaluated by SDS-PAGE using 4–12% gradient gels (Invitrogen, Carlsbad, CA). Endotoxin levels were quantified using the EndoZyme assay (bioMérieux, Nürtingen, Germany), and all antibody preparations exhibited endotoxin concentrations below 0.5 EU/mL.

### TCGA database analysis for NKG2DLs gene expression

Relative mRNA expression levels of *MICA*, *MICB*, and *ULBP1–4* in ovarian cancer tumor tissue samples were retrieved from The Cancer Genome Atlas (TCGA) and the Genotype-Tissue Expression (GTEx) project via the Gene Expression Profiling Interactive Analysis (GEPIA) web server.

### Cell lines and Peripheral Blood Mononuclear Cells (PBMCs)

Human ovarian cancer cell lines OVCAR-3, OVCAR-4, OVCAR-5, OVCAR-8, and NCI/ADR-RES were obtained from the American Type Culture Collection (ATCC) and maintained in Dulbecco’s Modified Eagle Medium (DMEM; Gibco) under standard culture conditions. Mycoplasma contamination was monitored every three months using standard detection protocols.

Peripheral blood mononuclear cells (PBMCs) were isolated from healthy donors of varying age and gender by density gradient centrifugation (Biochrom, Berlin, Germany). Donors were randomly selected for each experiment. Cryopreserved PBMCs were thawed and cultured overnight at 37 °C prior to use in functional assays. All donors provided written informed consent, and the study was conducted in accordance with the Declaration of Helsinki and approved by the local ethics committee.

### Polymerase Chain Reaction (PCR)

For analysis of *MICA*, *MICB*, and *ULBP1–4* mRNA levels, total RNA was extracted from 1 to 2 × 10⁶ ovarian cancer cells using the High Pure RNA Isolation Kit (Roche, Basel, Switzerland) according to the manufacturer’s instructions. cDNA synthesis was performed using FastGene Scriptase II (NIPPON Genetics Europe, Düren, Germany). Quantitative real-time PCR (qPCR) was subsequently carried out on a LightCycler 480 system (Roche) using PerfeCTa SYBR Green FastMix (Quanta Biosciences, Beverly, MA).

Gene-specific primers were employed to assess the expression levels of *MICA*, *MICB*, and *ULBP1–4*. The housekeeping gene *RPL13* (Hs_RPL13_1_SG; Qiagen) served as an internal control for normalization. Primer sequences and amplification conditions were used as previously described [38].

### Cell viability assays

To evaluate cell viability and metabolic activity, 10,000 ovarian cancer cells were seeded in each well and cultured for 72 h with or without the indicated constructs (10 µg/mL). After incubation, WST reagent (Roche) or the CellTiter-Glo^®^ Luminescent Cell Viability Assay (Promega, Madison, WI) was added to each well, and metabolic activity was measured according to the manufacturer’s instructions.

### Flow cytometry

Flow cytometric analysis was performed to assess the surface expression of individual NKG2DLs (MICA, MICB, ULBP1–4, and ULBP2/5/6) as well as total NKG2DLs expression. Staining was conducted using ligand-specific antibodies as previously described [38], followed by detection with a PE-conjugated anti-mouse secondary antibody (Jackson ImmunoResearch, West Grove, PA). Quantification of NKG2DLs surface molecules per cell was achieved using the QIFIKIT (Dako, Hamburg, Germany) in accordance with the manufacturer’s instructions.

For analysis of NK cell activation and degranulation, PBMCs were stained with fluorescently labeled antibodies targeting CD3 (APC/Fire), CD56 (PE-Cy), CD16 (APC), CD25 (BV711), and CD69 (PE) (all from BioLegend, San Diego, CA). Viability staining was performed using either 7-AAD (1:200 dilution; BioLegend) or LIVE/DEAD™ Fixable Aqua (Thermo Fisher Scientific) to exclude dead cells from analysis.

Samples were analyzed using either a BD FACS Canto II or BD LSRFortessa flow cytometer (BD Biosciences), and data were processed with FlowJo software (FlowJo LLC, Ashland, OR).

### Off-target assessment of NKG2D-ADCC in combination with chemotherapy

To evaluate potential off-target immune activation, PBMCs from healthy donors were incubated with carboplatin (0.36 µg/mL) for 24 h. Following this initial exposure, treatments (10 µg/mL each) or no treatment were added for an additional 24 h. NK cell activation within PBMCs was then assessed by measuring CD69 and CD25 expression by flow cytometry. NK cells were identified as CD3^−^ CD56^+^ cells within PBMCs.

### NK cell activation assays

To assess the activation of NK cells in healthy donor PBMCs, co-culture experiments were conducted with 30,000 ovarian cancer cells and PBMCs at an effector-to-target (E: T) ratio of 10:1 in the absence or presence of treatment (10 µg/mL). After 24 h, the cells were collected and labeled to evaluate CD69 and CD25 expression by flow cytometric analysis. NK cells were identified as CD3^−^ CD56^+^ cells within PBMCs.

### Analysis of cytokine secretion

To evaluate cytokine or immune effector molecule secretion, PBMCs from healthy donors were co-cultured with or without 30,000 ovarian cancer cells at an E: T ratio of 10:1, in the presence or absence of the indicated constructs (10 µg/mL). Following a 4-hour incubation period, culture supernatants were collected for cytokine/effector molecule analysis.

IFN-γ concentrations were quantified using an in-house sandwich ELISA assembled from validated individual components. High-binding 96-well plates were coated with anti-human IFN-γ mAb (M700A, Thermo Fisher Scientific) and blocked with 1% BSA/PBS. Samples and standards were incubated for 1–2 h at room temperature, followed by detection with a biotinylated anti-human IFN-γ mAb (M-701B, Thermo Fisher Scientific) and poly-HRP streptavidin (Thermo Fisher Scientific). TMB substrate (Thermo Fisher Scientific) was used for development, and reactions were stopped with 1 M H₃PO₄ before absorbance was measured at 450 nm.

For co-culture conditions involving target cells, secretion levels of IFN-γ, granzyme A, granzyme B, perforin, and granulysin were measured using LEGENDplex™ bead-based multiplex assays (BioLegend), in accordance with the manufacturer’s instructions.

### Cytotoxicity assays

The cytotoxic activity of PBMCs against ovarian cancer cells was assessed using BATDA Europium release assays (PerkinElmer, Waltham, MA) performed over a two-hour lysis period. Prior to co culture, target tumor cells were pre incubated with the respective fusion proteins or the isotype control for 30 min to allow sufficient surface binding. Following this pre incubation, PBMCs were added at the indicated effector to target ratios and incubated with the labeled target cells for two hours. Specific lysis was calculated according to the manufacturer’s instructions using the formula:

$$specific\:lysis=\:100\:\times\:\:\frac{\left[\left(experimental\:release\right)\:-\:\left(spontanous\:release\right)\right]}{\left[\left(maximum\:release\right)\:-\:\left(spontanous\:release\right)\right]}$$

To evaluate NK cell-mediated cytotoxicity using flow cytometry, ovarian cancer target cells were labeled with 2.5 µM CellTrace™ Violet (Thermo Fisher Scientific, Waltham, MA) and cultured with PBMCs from healthy donors at an E: T ratio of 10:1, with or without antibodies (10 µg/mL each). After incubating for either 24–72 h, the cells were stained with 7-AAD to determine viability. Assay volume consistency was controlled using counting beads (Sigma, St. Louis, MO). Target cell lysis was quantified by calculating the count of 7-AAD-negative, CellTrace-positive cells relative to the count in untreated controls.

To conduct long term analyses of cytotoxicity, 10,000 ovarian cancer cells were seeded in 96 well E plates and allowed to adhere and establish a stable baseline for 24 h. After this initial period, PBMCs from healthy donors were added at an E: T ratio of 10:1 together with the indicated constructs (5 µg/mL). Real time monitoring of the cell index was performed at 30-minute intervals for a 100-hour observation period using the xCELLigence RTCA system (Roche Applied Science, Penzberg, Germany). The time point of PBMC addition, which results in a visible inflection of the cell index curve, is indicated in the corresponding figure.

### Statistics

Unless otherwise specified, values are presented as means ± standard deviation (SD). Statistical analyses were performed using GraphPad Prism (version 10.1.1) and included one-way ANOVA and the Kruskal-Wallis test for continuous variables. If ANOVA revealed significant differences, a group-wise comparison was performed using the Tukey multiple comparisons test. If the Kruskal-Wallis test revealed significant differences, the Dunn’s multiple comparisons test was used. All statistical tests were considered significant when p was below 0.05.

## Results

### NKG2DLs in ovarian cancer

**Fig. 1:**
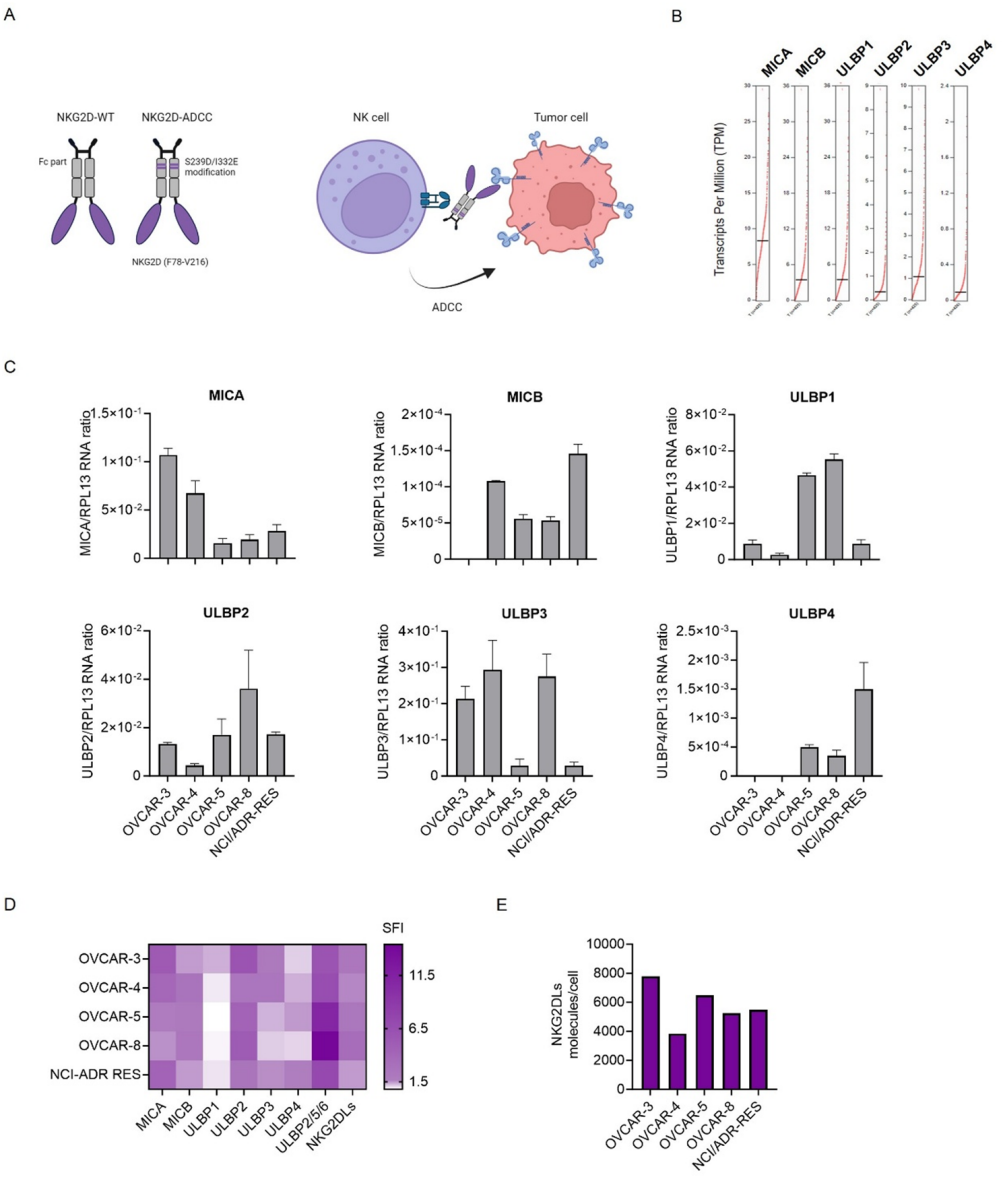
NKG2DLs Expression in ovarian cancer cells. **A** Schematic illustration and mechanism of action of the NKG2D-Ig fusion proteins. The graphic was created by BioRender (BioRender.com, Toronto, Canada). **B** Analysis of mRNA expression levels of NKG2DLs (MICA, MICB, ULBP1-4) in ovarian cancer using the online tool GEPIA. **C** Quantification of the indicated NKG2DL mRNA expression relative to RPL13 mRNA in five ovarian cancer cell lines (OVCAR-3, OVCAR-4, OVCAR-5, OVCAR-8, and NCI/ADR-RES). Data represent combined results from three independent experiments. **D** Surface expression of the indicated NKG2DLs on ovarian cancer cell lines, analyzed by flow cytometry. Cells were stained with mAbs against the respective NKG2DLs, either individually or as a cocktail, alongside appropriate isotype controls, followed by an anti-mouse PE-conjugated secondary antibody. SFI values were calculated as detailed in the Methods section. **E** Quantification of NKG2DLs molecules per cell on ovarian cancer cell lines by flow cytometry using the QIFIKIT assay. NKG2DLs: Ligands for the activating immune receptor NKG2D; PE: Phycoerythrin; SFI: Specific fluorescence intensity

In previous reports, NKG2D-IgG based fusion proteins have been described, which contain the extracellular domain of the NKG2D receptor fused to either a human IgG1 wild-type Fc portion (NKG2D-WT) or a human IgG1 portion harboring the amino acid substitutions S239D/I332E (NKG2D-ADCC). These substitutions are designed to enhance the affinity for the Fc receptor CD16, which mediates ADCC [39, 40]. Consequently, the functional activity of the fusion protein is improved (Fig. 1A). The expression of NKG2DLs has been reported in many solid tumors including ovarian cancer [13]. In the context of ovarian cancer tissue samples, as derived from TCGA data, relative expression of *MICA*, *MICB*, and *ULBP1-4* mRNA was observed (Fig. 1B). In subsequent analyses, we examined the levels of NKG2DLs mRNA in ovarian cancer cell lines OVCAR-3, OVCAR-4, OVCAR-5, OVCAR-8, and NCI/ADR-RES. The expression of *MICA*, *ULBP1*, *ULBP2*, and *ULBP3* was consistently detected across all ovarian cancer cell lines examined, with varying intensity. Furthermore, *MICB* mRNA was observed in four out of five cell lines, while *ULBP4* mRNA was detected in three out of five (Fig. 1C). Subsequently, the surface expression was determined using specific mAbs against MICA, MICB, ULBP1, ULBP2, ULBP3, and ULBP4. To analyze the expression of ULBP5 and ULBP6, an antibody recognizing both, together with ULBP2, was utilized. The detectable surface levels on the cell lines did not directly correlate with the mRNA expression levels determined by PCR analysis, consistent with the posttranslational regulation of NKG2DL expression [41]. ULBP2 and ULBP2/5/6 showed the highest prevalence and surface expression levels among the tested cell lines, whereas ULBP1 was undetectable (Fig. 1D, Supplemental Figure S1). The number of NKG2DL molecules ranged from 3830 (OVCAR-4) to 7771 (OVCAR-3) (Fig. 1E). To ensure that subsequent functional assays reflect the physiologically relevant variability of NKG2DLs expression observed across ovarian cancer models, we selected three representative cell lines (OVCAR-4, OVCAR-8 and OVCAR-5) that span the expression range detected in our panel.

### Fc optimization enhances binding to NK cells without inducing non-specific activation

**Fig. 2:**
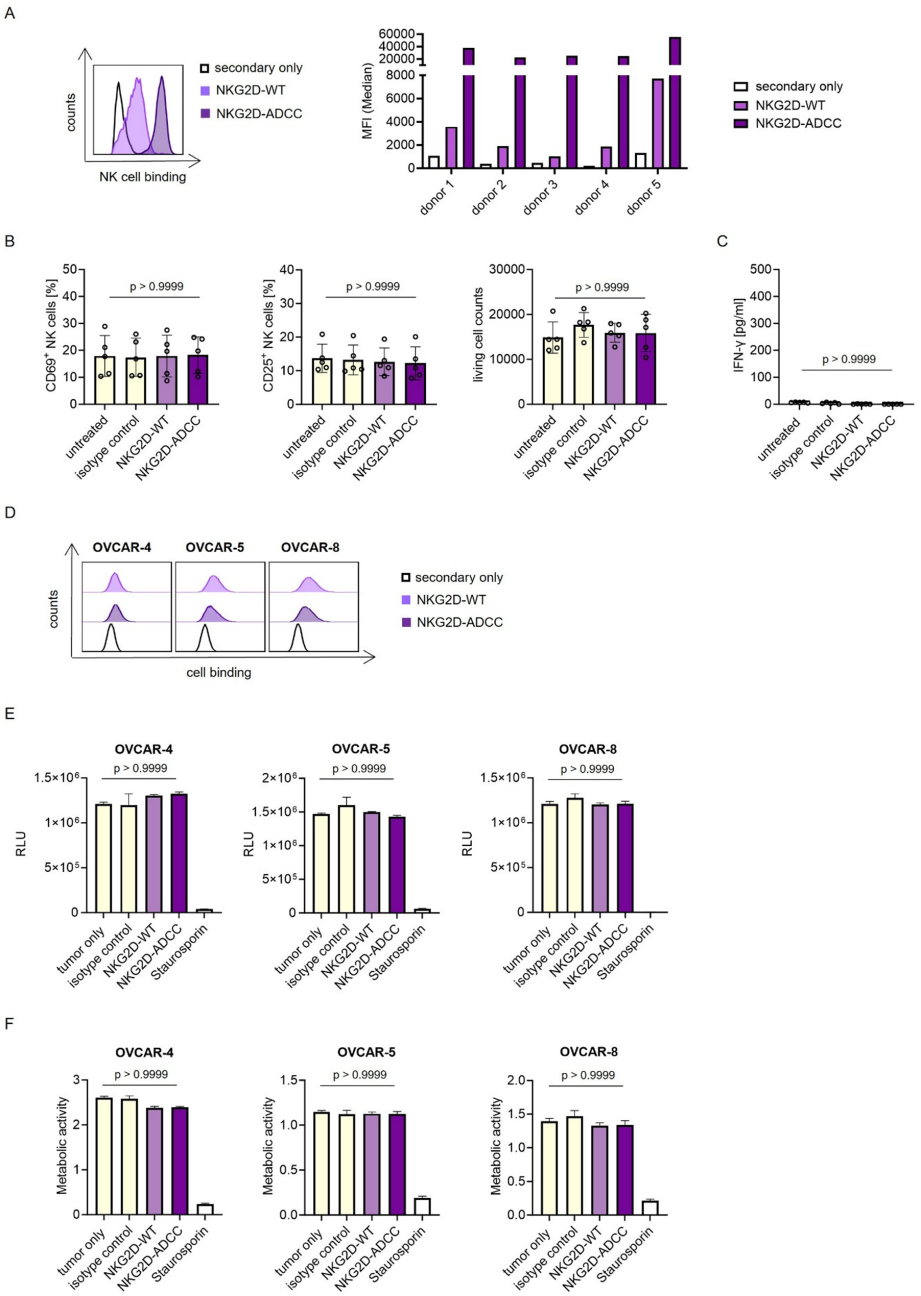
Characterization of binding and target cell-restricted efficacy of NKG2D-Ig fusion proteins. **A** PBMCs of healthy donors (n=5) were stained with the indicated NKG2D-Ig fusion protein, followed by an anti-human PE conjugate, and counterstained for CD56 und CD3. Binding of the fusion proteins to NK cells within PBMCs was analyzed by flow cytometry. The left panel shows representative histograms for a single donor (open peak: control; filled peaks: indicated fusion protein), the right graph displays MFIs for all five donors tested. **B**-**C** PBMCs of healthy donors (n=5) were cultured with or without the indicated treatment (10 µg/mL) for 24 h. **B** NK cell activation was assessed by flow cytometric analysis for CD69, CD25, as well as viable cell count. **C** IFN-γ levels in culture supernatants were measured using ELISA. **D** The indicated tumor cell lines were incubated with 10 µg/mL of the indicated NKG2D-Ig fusion proteins, followed by anti-human PE conjugate staining. Binding of the constructs to the cell lines was analyzed by flow cytometry. Open peaks depict the control, filled peaks depict fusion protein binding. **E**-**F** The indicated cells were treated with 10 µg/mL of the respective constructs for 72 h. **E** ATP levels were measured using CellTiterGlo assays. Representative data from one experiment out of at least two independent repeats are shown. **F** Metabolic activity was assessed by WST assays. Representative data from one experiment out of at least two independent repeats are shown. All conditions were measured in technical duplicates. MFI: mean fluorescence intensities; PBMCs: peripheral blood mononuclear cells; PE: phycoerythrin; ATP: adenosintriphosphat; WST: water-soluble tetrazolium salt 1

Subsequently, an assessment was performed to determine the binding capacity of both NKG2D-WT and NKG2D-ADCC to NK cells within PBMCs. While NKG2D-WT demonstrated detectable binding to NK cells, NKG2D-ADCC exhibited significantly enhanced binding, consistent with its engineered increased affinity for CD16 (Fig. 2A). To evaluate potential off-target effects of the fusion proteins in the absence of tumor cells, PBMCs were incubated with NKG2D-WT, NKG2D-ADCC, or their respective isotype control. The investigation revealed that neither NKG2D-targeting construct induced NK cell activation nor affected PBMCs viability. This finding suggests that these proteins do not elicit non-specific immune activation or cytotoxicity in the absence of target cells (Fig. 2B). Consequently, no IFN-γ secretion was detected in culture supernatants under these conditions (Fig. 2C). In addition, to evaluate potential off-target effects under conditions mimicking combination therapy, PBMCs were pretreated with a physiologically relevant dose of carboplatin. This resulted in only a slight, non-significant increase in NKG2DLs expression and did not meaningfully enhance NK cell activation by NKG2D-ADCC, supporting maintained target specificity (Supplemental Figure S2).

Binding analysis revealed comparable binding of both NKG2D-WT and NKG2D-ADCC to the ovarian cancer cell lines OVCAR-4, OVCAR-5, and OVCAR-8 (Fig. 2D). NKG2D-ADCC bound to all three cell lines in a dose-dependent manner (Supplemental Figure S3).

Given that certain therapeutic antibodies can mediate direct cytotoxic effects independent of immune effector cells, such as apoptosis induction by trastuzumab [42], we examined whether NKG2D-WT or NKG2D-ADCC exert similar effects. However, no reduction in cell viability (Fig. 2E) or metabolic activity (Fig. 2F) was observed in treated ovarian cancer cells compared to controls, indicating the absence of direct cytotoxicity by either fusion protein.

### Enhanced NK cell activation by NKG2D-ADCC in the presence of ovarian cancer cells

**Fig. 3:**
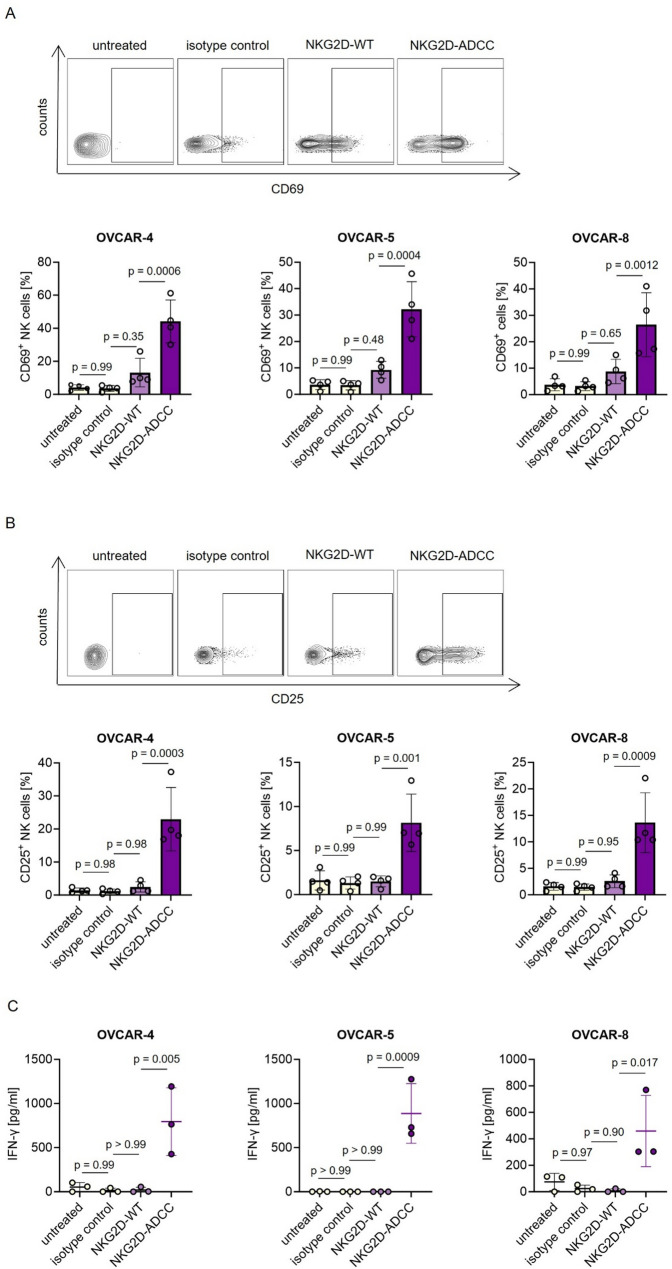
Induction of NK cell activation by NKG2D-Ig fusion proteins in co-culture with ovarian cancer cells. PBMCs from healthy donors were cultured with the indicated ovarian cancer cells at 10:1 E:T ratio with or without the NKD2D-Fc fusion proteins or corresponding isotype control (10 μg/mL). **A**, **B** NK cell activation was evaluated by flow cytometric analysis of (**A**) CD69 and (**B**) CD25 expression after 24 hours. Upper panels show representative dot plots from a single donor co-cultured with OVCAR-4 cells. Lower panels summarize combined data from four independent donors. **C** IFN-γ levels in culture supernatants were measured by ELISA after 24 hours. Data represent combined results from three independent donors. All conditions were measured in technical duplicates. E:T: effector to target; PBMCs: peripheral blood mononuclear cells

As NK cell activation constitutes a crucial initial step in the initiation of tumor cell lysis, we investigated the expression of CD69 as an early activation marker on NK cells in co-cultures of ovarian cancer cells and PBMCs. The gating strategy used to identify NK cells is shown in Supplemental Figure S4. Treatment with NKG2D-ADCC significantly increased CD69 expression on NK cells compared to both NKG2D-WT and isotype control, indicating robust activation (Fig. 3A). To further evaluate the functional capacity of NK cells following treatment, we analyzed the expression of CD25, a marker associated with sustained activation and cytotoxic potential [43]. Treatment with NKG2D-ADCC resulted in a pronounced upregulation of CD25 expression on NK cells, exceeding levels induced by NKG2D-WT and control conditions (Fig. 3B). These findings suggest that NKG2D-ADCC more effectively promotes a cytotoxic NK cell phenotype.

Consistent with this, we observed a significant increase in IFN-γ secretion upon treatment with NKG2D-ADCC (Fig. 3C). IFN-γ is a key effector cytokine involved in antitumor immunity, further emphasizing the enhanced functional activation of NK cells by the Fc-optimized construct. In parallel, we observed a donor-dependent but non-significant trend toward increased CD69 expression on T cells in the NKG2D-ADCC condition (Supplemental Figure S5), potentially reflecting secondary activation triggered by NK cell-derived cytokines such as IFN-γ [44]. Collectively, these results demonstrate that NKG2D-ADCC robustly promotes NK cell activation and functional responsiveness in the context of ovarian cancer.

### NKG2D-ADCC induces potent release of cytotoxic effector molecules by NK cells

ADCC is mediated by the release of immune effector molecules such as granzymes A and B, perforin, and granulysin - key mediators of target cell lysis. To assess the functional activation of NK cells, we measured the secretion of these cytotoxic proteins in co-cultures of PBMCs and ovarian cancer cells treated with either NKG2D-WT, NKG2D-ADCC, or an isotype control.

**Fig. 4:**
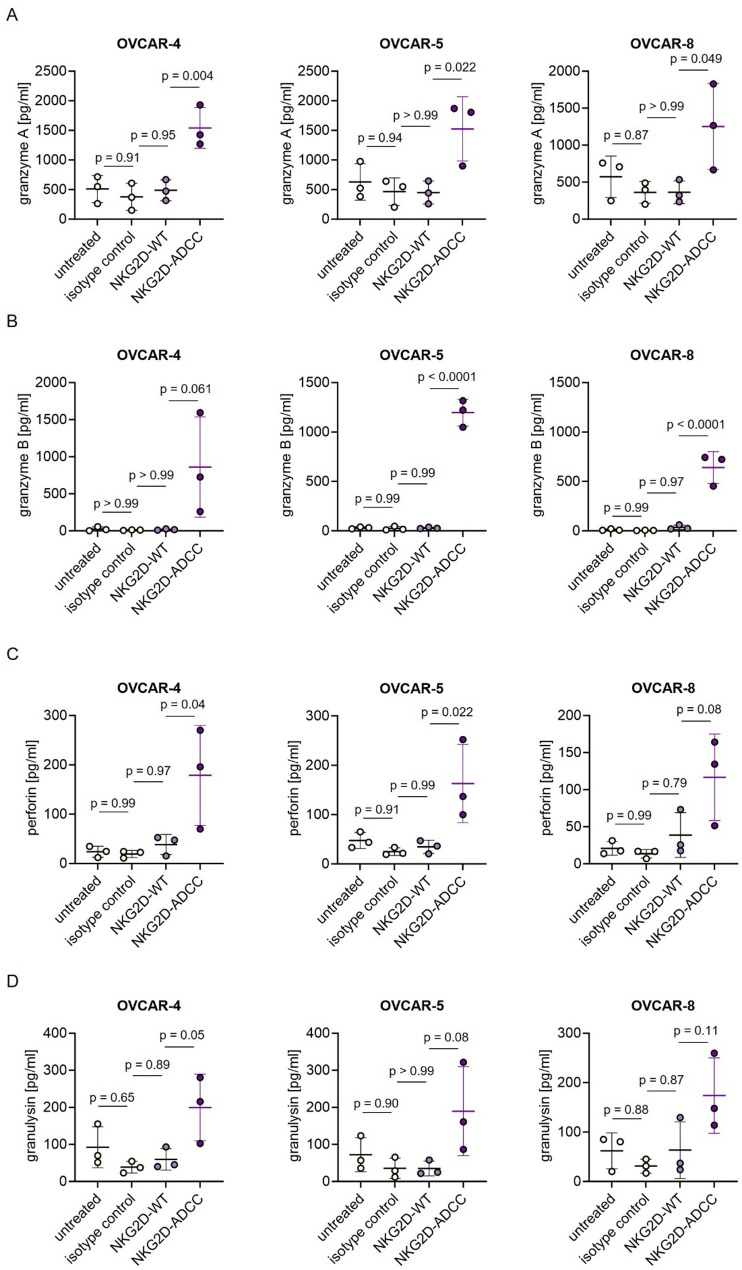
Induction of cytotoxic effector molecule release by NKG2D-Ig fusion proteins. PBMCs from healthy donors (n = 3) were co-cultured with the indicated ovarian cancer cells at a 10:1 E:T ratio in the presence or absence of NKG2D-Ig fusion proteins or isotype controls (10 µg/mL). After 6 hours, levels of (**A**) granzyme A (**B**) granzyme B , (**C**) perforin, and (**D**) granulysin were quantified in culture supernatants using LEGENDplex assays. All conditions were measured in technical duplicates. E:T: effector to target; PBMCs: peripheral blood mononuclear cells

Treatment with NKG2D-ADCC resulted in a significantly elevated release of granzyme A (Fig. 4A), granzyme B (Fig. 4B), perforin (Fig. 4C), and granulysin (Fig. 4D) compared to both NKG2D-WT and isotype control. Importantly, none of these effector molecules were released in response to the isotype control, highlighting the target cell-restricted and NKG2DLs-specific mode of action of the NKG2D-ADCC fusion protein. These findings further confirm the capacity of NKG2D-ADCC to robustly trigger the cytotoxic machinery of NK cells in a tumor-directed manner.

### NKG2D-ADCC effectively induces potent and sustained lysis of ovarian cancer cells

**Fig. 5:**
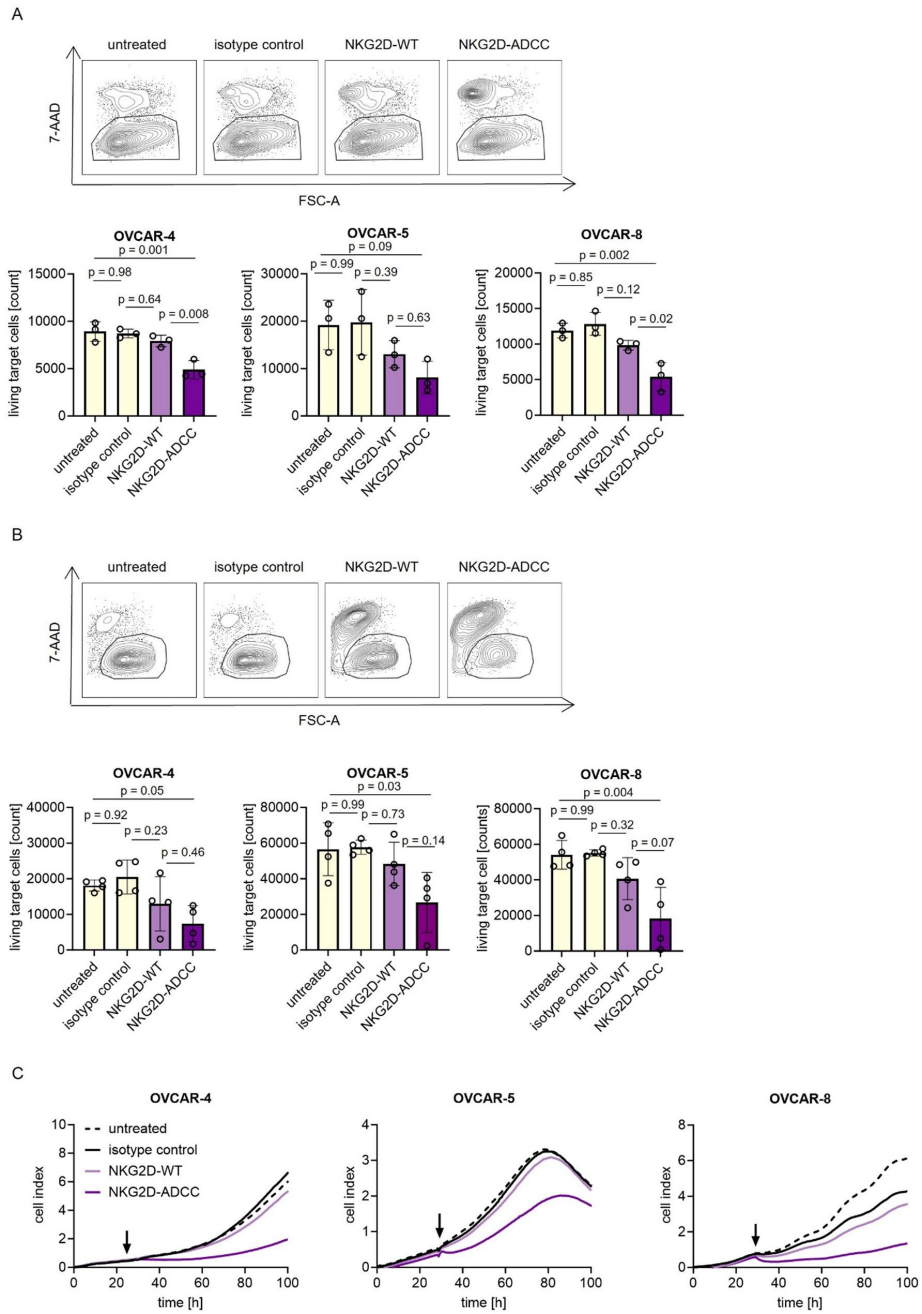
NKG2D-Ig fusion proteins induce lysis of ovarian cancer cell lines. PBMCs from healthy donors were incubated with the indicated target cells at an E:T ratio of 10:1 in the presence or absence of indicated treatments (10 µg/mL). **A**, **B** Tumor cell lysis was measured by flow cytometry-based assays after (**A**) 24 hours and (**B**) 72 hours of co-culture. **C** Long-term cytotoxicity was assessed using the xCELLigence system. For these assays, PBMCs and target cells were co-cultured at an E:T ratio of 10:1 in the presence or absence of 5 µg/mL NKG2D-WT, NKG2D-ADCC, or the corresponding isotype control. The time point of PBMCs addition (24 hours) is indicated by arrows in the plot. All conditions were measured in technical duplicates. E:T: effector to target; PBMCs: peripheral blood mononuclear cells

Given the robust NK cell activation and effector molecule release observed following NKG2D-ADCC treatment, we next evaluated whether these responses translated into effective tumor cell killing. NK cell-mediated cytotoxicity is regulated by the integration of activating and inhibitory signals, with engagement of CD16 via Fc interactions known to elicit rapid and potent responses. Rapid induction of tumor cell lysis by NKG2D-ADCC was observed in short-term Europium-based cytotoxicity assays (Supplemental Figure S6). To further assess the cytolytic efficacy of our fusion proteins, flow cytometry-based tumor cell lysis assays were conducted after 24 h of co-culture with PBMCs. Treatment with NKG2D-ADCC resulted in significantly enhanced tumor cell lysis compared to the wildtype Fc fusion protein (NKG2D-WT), which demonstrated only modest effects (Fig. 5A). Quantitatively, NKG2D-ADCC induced on average 54% lysis in OVCAR-4, 59% in OVCAR-5, and 55% in OVCAR-8 at the 24-hour time point. These findings were further supported by the 72-hour assays, in which NKG2D-ADCC continued to mediate substantial tumor cell killing, reaching 60% in OVCAR-4, 52% in OVCAR-5, and 70% in OVCAR-8 (Fig. 5B). Moreover, long-term killing was confirmed using real-time impedance-based assays, revealing sustained and potent lysis of ovarian cancer cells exclusively in the presence of NKG2D-ADCC, whereas NKG2D-WT showed markedly reduced efficacy (Fig. 5C). Importantly, no cytotoxicity was detected under isotype control conditions in any of the cytotoxicity assays, underscoring the target-specific mode of action.

In summary, NKG2D-ADCC effectively translates NK cell activation into robust and durable tumor cell killing, highlighting its strong therapeutic potential for targeting NKG2DL-expressing ovarian cancer.

## Discussion

Despite advances in immunotherapy across multiple solid tumors, ovarian cancer remains a major clinical challenge due to its frequent late-stage diagnosis, high relapse rates, and common resistance to conventional chemotherapy [45]. In this study, we present a novel Fc-optimized NKG2D-Ig fusion protein (NKG2D-ADCC) that exploits the tumor-restricted expression of NKG2DLs to direct and enhance NK cell-mediated cytotoxicity against ovarian cancer cells. Our data demonstrate that NKG2D-ADCC effectively bridges NK cells to ovarian cancer targets by recognizing a diverse repertoire of NKG2DLs while simultaneously engaging CD16 to activate NK cell effector functions.

A key advantage of this approach lies in the broad ligand recognition capability of the NKG2D receptor, which binds structurally diverse ligands including MICA, MICB, and members of the ULBP family [1]. These ligands are commonly upregulated in various tumor types, including ovarian cancer [11]. Unlike mAbs, typically restricted to a single epitope or antigen, the NKG2D-ADCC fusion protein retains functional versatility by targeting multiple ligands, thereby potentially mitigating immune escape mechanisms such as antigen loss or downregulation. Our findings confirm that ovarian cancer cell lines consistently express at least two NKG2DLs at the protein level, reinforcing the rationale for this multi-targeting strategy.

A well-recognized immune escape mechanism of NKG2DLs-expressing tumors is the proteolytic shedding of MICA, MICB and several ULBP family members, which can reduce surface ligand density, generate soluble ligand “sinks,” and downregulate NKG2D on effector cells [46–48]. This process is therefore an important consideration for NKG2D-based therapeutics. In our study, NKG2D-ADCC remained functionally active across all tested ovarian cancer models despite their known capacity for ligand shedding, indicating that sufficient surface ligand is retained to support NK cell engagement. Nevertheless, soluble NKG2DLs may influence therapeutic efficacy in vivo. Strategies aimed at limiting ligand shedding, such as inhibition of ADAM proteases or combination with shedding-blocking MICA/B antibodies, may thus further enhance the activity of NKG2D-ADCC [49, 50]. Future studies will be required to define the extent to which soluble ligands affect pharmacodynamics and to explore rational combination approaches to overcome this mechanism.

Another aspect of the NKG2D/NKG2DLs axis that warrants consideration is the regulation of endogenous NKG2D expression in the presence of soluble or shed ligands. Previous studies have shown that high or sustained levels of soluble MICA and MICB can reduce NKG2D surface expression on NK cells and CD8 T cells and impair cytotoxic function [51]. Tumor derived exosomes carrying NKG2DLs in combination with TGF-β have similarly been reported to downregulate NKG2D on effector lymphocytes under prolonged exposure conditions [52]. Reviews of the pathway highlight that such effects predominantly occur in settings characterized by persistent or high level ligand exposure within the tumor microenvironment [53]. Whether NKG2D based fusion proteins influence this process, for example by binding soluble ligands, remains an open question. Future mechanistic studies designed to model sustained ligand exposure together with longitudinal analysis of effector cell subsets will be required to clarify how endogenous NKG2D expression is modulated in this context.

By introducing the SDIE mutations into the Fc domain, we enhanced the binding affinity of the fusion protein to the Fcγ receptor CD16, thereby augmenting NK cell activation and ADCC. Compared to the wildtype Fc variant, NKG2D-ADCC induced significantly stronger NK cell activation, as evidenced by elevated expression of activation markers CD69 and CD25, IFN-γ secretion, as well as enhanced release of cytotoxic effectors including granzyme A/B, perforin, and granulysin. Furthermore, both short- and long-term cytotoxicity assays demonstrated that this immune activation translated into potent and sustained lysis of ovarian cancer cells. Importantly, control fusion proteins with irrelevant specificity failed to induce similar responses, underscoring the specificity of NKG2D-ADCC-mediated engagement. In future studies, the functional profiling could be further expanded by including CD107a degranulation assays, which represent an established readout of NK cell cytotoxic activity and would complement the activation and cytotoxicity markers assessed here. At present, no conclusions can be drawn regarding how NKG2D-ADCC compares with established mAb-based approaches, as this was not addressed in the current study. Future work will be required to experimentally assess how NKG2D-based targeting performs relative to clinically used antibodies and to define in which therapeutic contexts this strategy may offer complementary benefit.

NK cells have gained interest as a therapeutic avenue in ovarian cancer due to the inherent immunogenicity of the disease [35, 54]. Tumor-infiltrating lymphocytes, comprising both T cells and NK cells, are frequently detected in ovarian tumor tissues, and their presence correlates with improved clinical outcomes [55]. While the precise role of NK cells in patient prognosis remains to be fully elucidated, preclinical studies consistently demonstrate the susceptibility of ovarian cancer cells to NK cell-mediated cytotoxicity [34]. These observations support the rationale for developing NK cell-based therapeutic strategies to improve clinical outcomes in ovarian cancer.

Our data further highlight the potential of NKG2D-ADCC as a complementary or alternative approach to existing NKG2D/NKG2DLs-based therapies, such as NKG2D-CAR T or CAR NK cells, which have shown efficacy in preclinical models of ovarian and other cancers [56]. While promising, cellular therapies are often limited by manufacturing complexity, high cost, and risks of severe toxicities, including cytokine release syndrome. In contrast, the NKG2D-ADCC fusion protein represents an “off-the-shelf”, scalable biologic with defined pharmacokinetics, controllable dosing, and reduced logistical challenges. Moreover, its format allows for flexible engineering to fine-tune its pharmacodynamic properties or incorporate additional immune-modulatory features, e.g. by introducing amino acid modification into the Fc part. Although we have not yet determined pharmacokinetic parameters for NKG2D-ADCC, its IgG1 Fc based format is expected to confer an extended serum half-life through FcRn mediated recycling. Therapeutic fusion proteins that combine an extracellular receptor domain with a human IgG1 Fc region typically display serum half-lives of several days in preclinical models and can reach several days to more than one week in clinical use, as reported for approved receptor Fc fusion biologics such as etanercept, abatacept or belatacept [57–60]. These data provide a general framework for the expected pharmacokinetic behavior of this class of molecules and support the assumption that NKG2D-ADCC will likewise show prolonged systemic exposure.

Given that NKG2DLs expression can be upregulated in response to chemotherapeutic agents or radiation [61], NKG2D-ADCC may be particularly effective when combined with standard-of-care treatments. For example, DNA damage-inducing agents or HDAC inhibitors have been shown to elevate NKG2DLs expression, potentially enhancing tumor cell susceptibility to NKG2D-targeted approaches [62]. Recently, an IND application for a mAb that inhibits MICA/B shedding was accepted by the FDA to enable initiation of a first-in-human clinical trial, highlighting a promising strategy to augment NK cell-mediated tumor cytotoxicity [63]. Combining such approaches with NKG2D-ADCC treatment could provide synergistic benefits. Furthermore, combination with immune checkpoint inhibitors may help overcome NK cell exhaustion or immunosuppressive tumor microenvironments that limit the efficacy of monotherapy. These potential synergies warrant further preclinical investigation.

While our findings support the therapeutic potential of NKG2D-ADCC, several critical questions remain. Although NKG2DLs expression is generally restricted to malignant tissues, it can be transiently induced in normal tissues during inflammation or infection. Therefore, in vivo studies are essential to assess the safety profile of NKG2D-ADCC and its potential for off-tumor toxicity. Utilization of humanized mouse models or patient-derived xenografts will be important for evaluating therapeutic efficacy, pharmacokinetics, and immune activation within physiologically relevant contexts [64–66]. A major limitation of our study is the lack of in vivo data, which will be the next focus of our work.

Additionally, biomarker-driven strategies to stratify patients based on NKG2DLs expression may enhance the clinical utility of NKG2D-ADCC. Given the heterogeneous expression observed in ovarian cancer cell lines, companion diagnostics could help identify patients most likely to benefit from this therapy. Future efforts might also focus on optimizing the fusion protein’s half-life or immunogenicity profile to further improve translational readiness.

In summary, this study provides preclinical evidence that Fc-optimized NKG2D fusion proteins can selectively and potently engage NK cells to mediate ADCC against ovarian cancer cells. This strategy exploits the natural tumor-recognition capabilities of the NKG2D receptor combined with Fc engineering to enhance immune activation.

Taken together, our results offer a compelling preclinical rationale for the development of Fc-engineered NKG2D fusion proteins as a versatile and tumor-specific immunotherapeutic platform with broad applicability for ovarian cancer and other NKG2DLs-expressing malignancies.

## Supplementary Information

Supplementary Material 1.

## Data Availability

The datasets generated during and/or analyzed during the current study are available from the corresponding author on reasonable request.
