# Supplementary material for: Targeting NKG2DLs with an ADCC enhanced fusion protein for induction of NK cell reactivity against ovarian cancer
Source: J Ovarian Res. 2026 Jan 21;19:59. doi: 10.1186/s13048-025-01962-2 (PMC12906047; doi:10.1186/s13048-025-01962-2)
Supplement: Supplementary file 1 — Supplementary Material 1. [file 13048_2025_1962_MOESM1_ESM.docx]

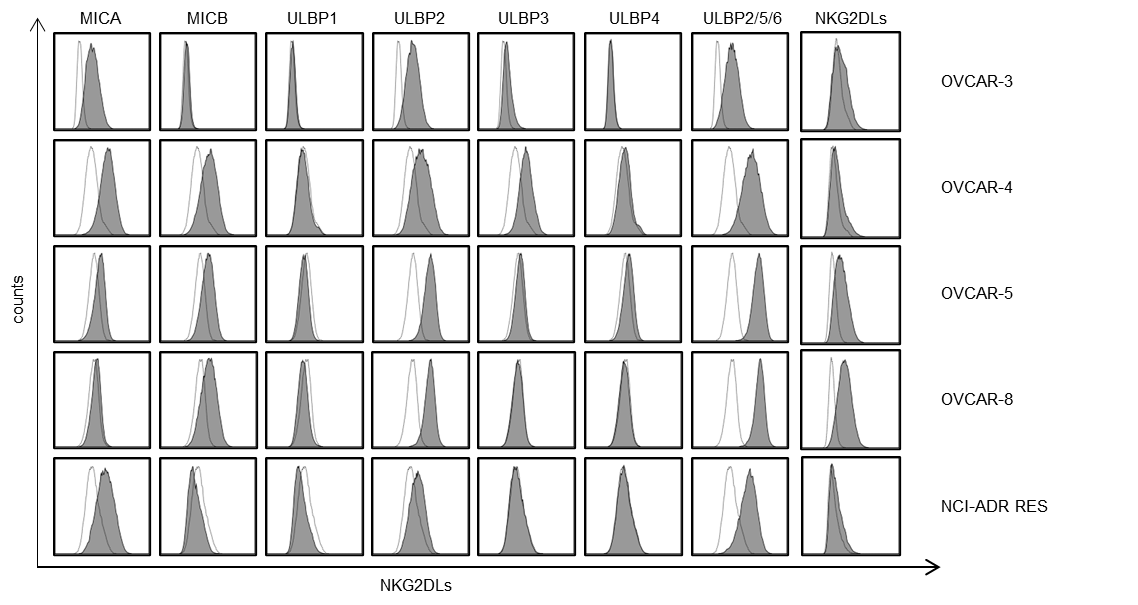


**Figure S1: Expression of NKG2DLs on ovarian cancer cell lines.** The surface expression of MICA, MICB, ULBP1-4, and ULBP2/5/6 was analyzed by flow cytometry on the indicated cell lines. MAbs against the depicted NKG2DLs are shown as shaded peaks; corresponding isotype controls are represented by open peaks.


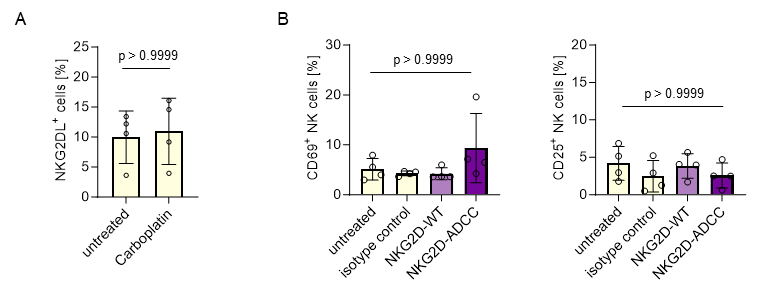


**Figure S2: Off-target assessment of NKG2D-ADCC in combination with chemotherapy.** PBMCs from healthy donors (n = 4) were incubated with 0.36 µg/ml carboplatin. **A**: After 24 hours, surface expression of NKG2D ligands was analyzed by flow cytometry. **B**: Following 24 hours of carboplatin exposure, the indicated treatments (10 µg/ml each) were added for an additional 24 hours. Activation of NK cells within PBMCs was then assessed by measuring CD69 and CD25 expression by flow cytometry.


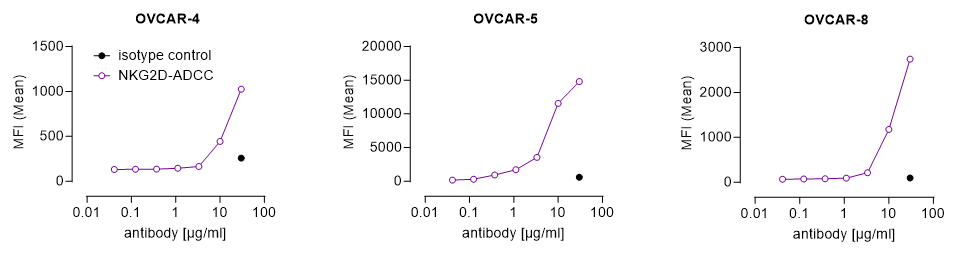


**Figure S3: Binding of NKG2D-ADCC to ovarian cancer cell lines.** The tumor cells were incubated with increasing concentrations of NKG2D-ADCC or the corresponding isotype control, followed by an anti-human PE conjugate. Binding of the constructs to the indicated cell lines was analyzed by flow cytometry. Data represent one representative experiment out of three independent repeats. MFI: mean fluorescence intensity.


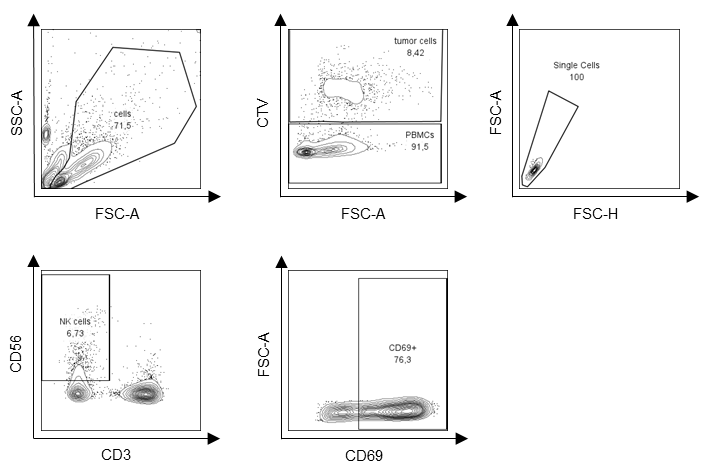


**Figure S4: Exemplary gating strategy used for NK cell analysis.** PBMCs from healthy donors were cultured with the indicated ovarian cancer cell lines at an E:T ratio of 10:1 in the presence or absence of the NKG2D Fc fusion proteins or the corresponding isotype control (10 µg/ml). NK cell activation was assessed by flow cytometric analysis of CD69 expression. The gating strategy consisted of sequential selection of cells by FSC A and SSC A, exclusion of CellTrace violet positive tumor cells, identification of single cells by FSC H and FSC A, and subsequent gating on NK cells defined as CD3 negative CD56 positive lymphocytes. Activated NK cells were identified as CD69 positive cells within this population.


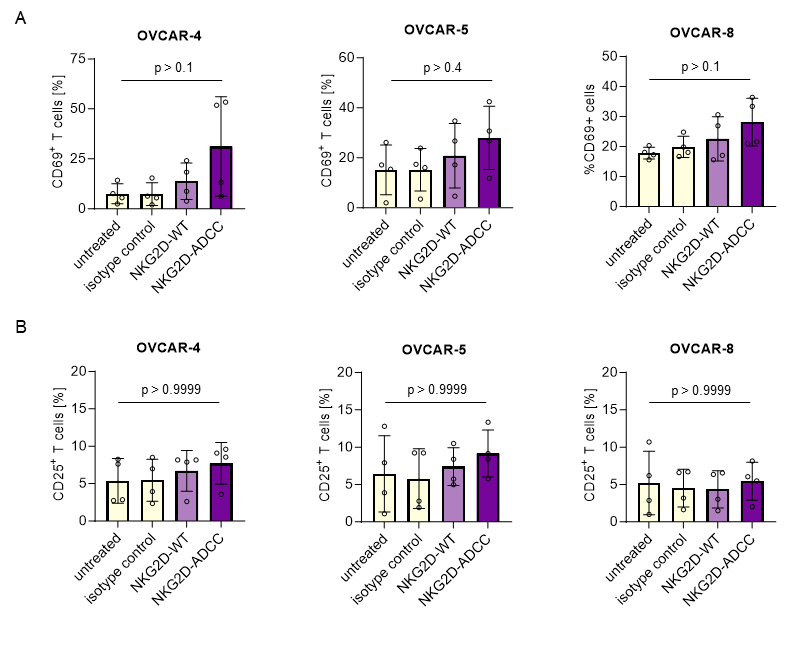


**Figure S5: effects on T cell activation by NKG2D-ADCC.** PBMCs from healthy donors (n = 4) were cultured with the indicated ovarian cancer cells at 10:1 E:T ratio with or without the NKD2D-Fc fusion proteins or corresponding isotype control (10 μg/mL).**(A, B)**T cell activation (CD3^+^ cells) was evaluated after 72 hours by flow cytometric analysis of **(A)** CD69 and **(B)** CD25 expression.

All conditions were measured in technical duplicates. E:T: effector to target; PBMCs: peripheral blood mononuclear cells.


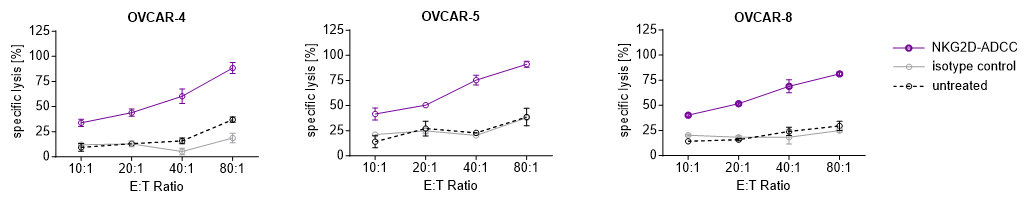


**Figure S6: Induction of target cell lysis by NKG2D-ADCC.** PBMCs from healthy donors were incubated with the indicated cell lines and treated with the indicated constructs (2.5 µg/mL). Ovarian cancer cell lysis was analyzed using 2-hour Europium cytotoxicity assays. Data represent one exemplary donor, measured in technical triplicates; error bars indicate standard deviation (SD). Exemplary data obtained with different E:T ratios are shown. E:T: effector to target; PBMCs: peripheral blood mononuclear cells.
